# Unraveling the Genetic Tapestry: A Case Report on Oro-Facial-Digital Syndrome’s Rare Features Across Generations in a Familial Trilogy

**DOI:** 10.7759/cureus.56623

**Published:** 2024-03-21

**Authors:** Nirat Tiwari, Keta Vagha, Aaditi Agarwal, Punam Uke, Ashish Varma, Sri Sita Naga Sai Priya K

**Affiliations:** 1 Pediatrics, Jawaharlal Nehru Medical College, Datta Meghe Institute of Higher Education & Research, Wardha, IND

**Keywords:** case report, inherited disorder, oro-facial digital syndrome, mohr syndrome, syndactyly, polydactyly

## Abstract

Oro-facial-digital syndrome, specifically Mohr syndrome, is an uncommon genetic disorder characterized by predominant oro-facial anomalies and polysyndactyly. While typically associated with autosomal recessive and X-linked dominant inheritance patterns, this case presents an autosomal dominant mode of transmission. This report documents the clinical presentation of three individuals, a 12-year-old male child and two females, 10-year-old and eight-year-old, who have inherited the disorder from their ancestors. The observed features include post-axial polysyndactyly in both upper and lower limbs, with the male child exhibiting additional manifestations of strabismus and knee joint defects. Symptomatic management is pursued due to the absence of complications, with surgical interventions and subsequent cosmetic repairs planned for all three children. Post-surgical physiotherapy is scheduled as part of their comprehensive treatment plan. The prognosis for this disorder is generally favorable, with a complete recovery anticipated and no complications expected.

## Introduction

Oro-facial-digital syndrome (OFDS) type 2, commonly known as Mohr syndrome, is a rare genetic disorder affecting the normal development of facial and oral anatomy, accompanied by post-axial polydactyly [1]. Typically inherited through autosomal recessive and X-linked dominant patterns, the presented case reveals an unusual autosomal dominant mode of inheritance, contributing to its rarity [2]. The study involves a 12-year-old male child and two females, a 10-year-old and an eight-year-old, exhibiting post-axial polysyndactyly and dental overcrowding. The male child additionally presented with strabismus and knee joint defects. OFDS patients may manifest other oro-facial abnormalities such as a bifid nose, cleft lip, and palate, strabismus, a bifid tongue, or a flat nasal bridge, and may experience organ dysfunctions later in life, including cardiovascular and renal anomalies and cerebral malformations impacting the intelligence quotient [3]. The incidence of OFDS is reported at 1 in 50,000 to 1 in 250,000 live births [4].

Historical inquiry reveals a familial pattern, with the patient’s grandfather and father also experiencing similar symptoms, albeit with lower severity. OFDS presentations vary, necessitating individualized treatment based on clinical symptoms. While infants with manifest dominant genetic disorders may face life-threatening challenges, the patient in this report experienced manageable complications. Surgical and cosmetic interventions were employed to address polysyndactyly in both hands. The prognosis in most OFDS cases is favorable, although lethality is possible in certain instances.

This case report delves into the diverse signs, symptoms, and management approaches for patients, emphasizing the societal outcome and viability of individuals affected by OFDS.

## Case presentation

Case 1

**Figure 1:**
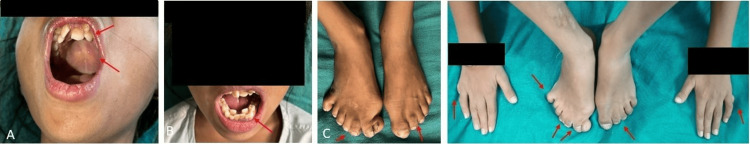
Case 1: (A) showing a high arched palate, (B) showing overcrowding of teeth, (C) showing polydactyly in the right foot and syndactyly between the second and third digits in both feet, and (D) showing post-axial polysyndactyly in both hands and feet

A 10-year-old female child was admitted to the hospital accompanied by her grandparents, presenting with a complaint of post-axial polysyndactyly in both the right and left hands and feet since birth. Upon examination, the patient exhibited an additional digit of approximately 1 cm in each hand and two extra digits on the right foot. Syndactyly was observed between the second and third digits in both the right and left feet. Additionally, the patient displayed dental overcrowding and a high-arched palate (Figure 1).

**Figure 2:**
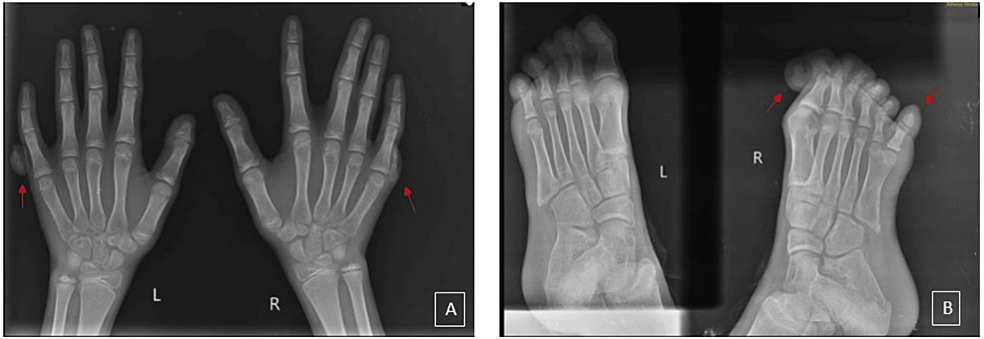
Case 1: X-rays of hands and feet confirming polydactyly

The physical examination revealed no other abnormalities. Vital signs were within normal limits, with a heart rate of 82 beats per minute, a respiratory rate of 24 per minute, a blood pressure of 110/60 mm Hg, and an oxygen saturation of 99%. The patient had achieved all developmental milestones for her age and lacked comorbidities or associated defects; therefore, surgical excision of the supernumerary digits was planned. X-rays of the right and left hands and feet confirmed the appropriate surgical sites (Figure 2).

**Figure 3:**
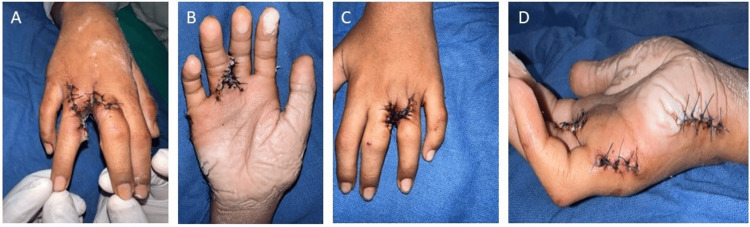
Case 1: (A) and (B) showing surgical correction of polysyndactyly in the right hand and (C) and (D) showing surgical correction of polysyndactyly in the left hand

Further investigations, including ultrasonography of the abdomen, echocardiography, and ophthalmological examination, showed no anomalies. This patient underwent syndactyly release and web space creation under general anesthesia (Figure 3).

The surgery was uneventful, followed by postoperative management with broad-spectrum antibiotics and analgesics.

Case 2

**Figure 4:**
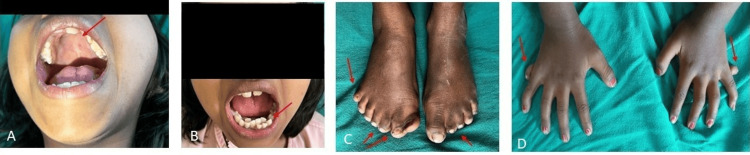
Case 2: (A) showing a high arched palate, (B) showing overcrowding of teeth, (C) showing polydactyly in the right foot and syndactyly in the second and third digits of both feet and the great toe of the right foot, and (D) showing polydactyly in both hands

An eight-year-old female child presented to the hospital with complaints of post-axial polysyndactyly in both hands and feet since birth. Examination revealed one additional digit of approximately 1 cm in both the right and left hands and two extra digits on both the right and left feet, with syndactyly between the second and third digits. The patient also displayed dental overcrowding and a high-arched palate (Figure 4).

**Figure 5:**
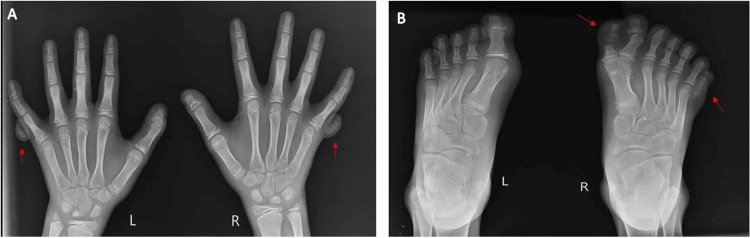
Case 2: (A) and (B) showing X-rays of the right and left hands and feet, confirming polydactyly

Vital signs were within normal limits, with a heart rate of 80 beats per minute, a respiratory rate of 24 per minute, a blood pressure of 100/60 mm Hg, and an oxygen saturation of 100%. X-rays of the right and left hands and feet were performed (Figure 5), and surgical intervention of syndactyly release and web space creation has been planned for the patient subsequently. Additional investigations, including ultrasonography of the abdomen, echocardiography, and ophthalmological examination, showed no anomalies.

Case 3

**Figure 6:**
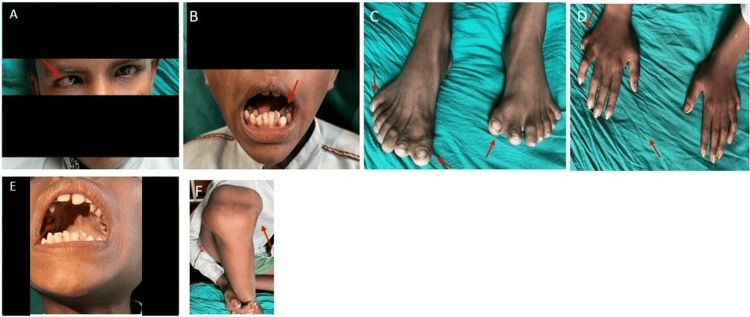
Case 3: (A) showing strabismus in the right eye, (B) showing overcrowding of teeth, (C) showing polydactyly in both feet and syndactyly in both great toes, (D) showing polydactyly in both hands, (E) showing high arched palate, and (F) showing knee joint deformity in the right leg

A 12-year-old male child was brought to the hospital with complaints of post-axial polysyndactyly in both hands and feet since birth. The examination revealed one additional digit in each hand and two extra digits in each foot. The patient also presented with strabismus in the right eye since birth, dental overcrowding, a high-arched palate, and a knee deformity that did not limit normal movement (Figure 6).

No comorbidities were identified, and the patient had achieved all developmental milestones for his age. Vital signs were within normal limits, with a heart rate of 80 beats per minute, a respiratory rate of 20 per minute, a blood pressure of 100/60 mm Hg, and an oxygen saturation of 98%. X-rays confirmed the diagnosis, and surgical intervention of syndactyly release and web space creation has been planned for the patient subsequently. Investigations, including ultrasonography of the abdomen, echocardiography, and ophthalmological examination, showed no anomalies.

**Figure 7:**
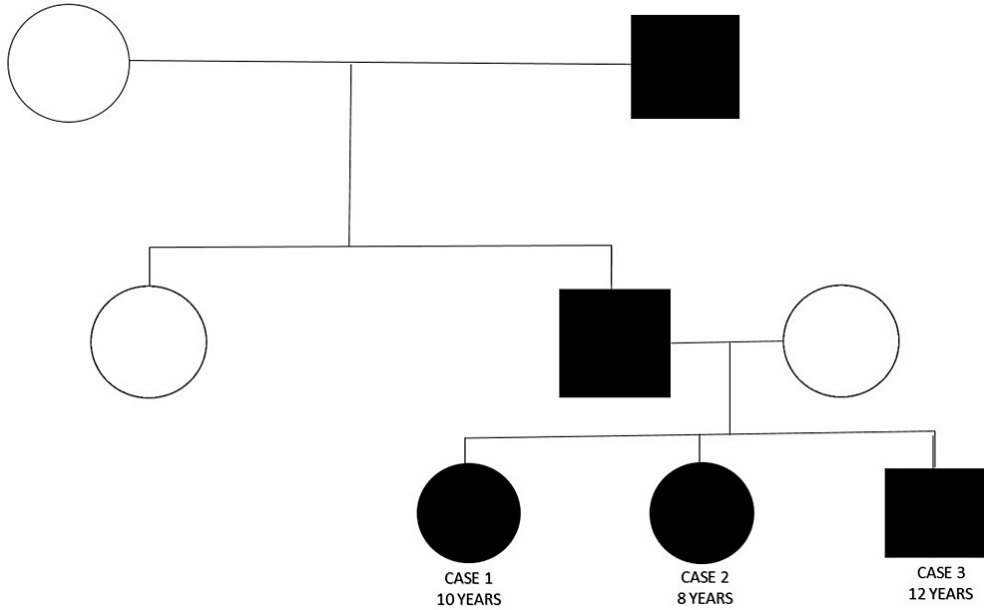
Pedigree chart of Case 1, Case 2, and Case 3 showing an autosomal dominant mode of inheritance

According to the eldest child, similar complaints of post-axial polysyndactyly are evident in the digits of the hands and feet of their father and paternal grandfather. The grandfather also had strabismus in his right eye. The pedigree chart in Figure 7 illustrates the affected family members and the autosomal dominant mode of inheritance of the disease. On tracing the patient’s history, the grandfather of the paternal side and the father, including Case 1, Case 2, and Case 3, were the affected members. The patients were advised to undergo genetic testing for the NEK1 and OFD2 gene defects, but due to their low financial status, their guardians refused to do so.

Patients with OFDS are managed symptomatically. However, life expectancy is low in cases with severe systemic abnormalities. The reported cases lack severe organ defects, and consequently, the prognosis for the genetic defect is favorable.

## Discussion

OFDS encompasses a collection of rare disorders characterized by diverse signs and symptoms that prominently affect the oral, facial, and digital aspects of individuals. The study involves a 12-year-old male child, a 10-year-old female, and an eight-year-old female exhibiting post-axial polysyndactyly and dental overcrowding. The male child additionally presented with strabismus and knee joint defects. Facial abnormalities may include hair thickening, hypertelorism, a flat nasal bridge, strabismus, and defects in the nasal septum. Oral manifestations may involve cleft lip and palate, bifid or nodular tongue, speech disability, and supernumerary dentition. Limb abnormalities encompass post-axial polydactyly, syndactyly, and skeletal malformations affecting joint movement. Other potential defects include hearing loss, incomplete brain development, and cardiovascular anomalies that aid in the differentiation of type 2 OFDS from other variations [2].

With 14 different types of OFDS, each exhibiting distinct inheritance patterns and clinical presentations, differentiation becomes challenging. This report focuses on type 2 (Mohr syndrome), a rare instance of autosomal dominant inheritance. Type 1 (Papillon-Léage-Psaume syndrome) is an X-linked dominant ciliopathy; type 3 (Sugarman syndrome) is an autosomal recessive gene defect; and various other types present autosomal recessive inheritance with diverse systemic abnormalities [3].

Despite the similarities in inheritance patterns and clinical features among the OFDS types, specific genetic investigations are crucial for an accurate diagnosis. The described cases detail symptoms such as thick hair, strabismus, dental overcrowding, post-axial polydactyly, and skeletal deformities [4]. Familial connections reveal similar features across generations. The autosomal dominant mode of inheritance observed in this report is attributed to consanguineous marriage, leading to a mutation in both parental carriers and manifesting as a dominant feature in the offspring [5].

The genetic mutation involves chromosomes X and 4q33, with a compound heterozygous mutation in the OFD2 and NEK1 genes linked to OFDS. OFDS management focuses on the symptomatic correction of oro-facial and limb abnormalities, prioritizing severe complications for survival [6]. Surgical interventions, reconstructive surgeries, and cosmetic repairs are employed, along with special training and physiotherapy for disabilities. Patients typically recover fully, with counseling provided for future disease outcomes. OFDS type 1 is considered more lethal, with a prognosis correlating with the severity of deformities and systemic malfunctions [3].

OFDS predominantly manifests within populations characterized by low socioeconomic status, where consanguineous marriages are prevalent. This prevalence underscores the persistence of recessive and remote genetic defects within society, contributing to the perpetuation of defective genes in the community [1]. In India, a society that places considerable importance on understanding individuals’ castes before marriage, genetic defects have the potential to span across numerous generations.

## Conclusions

OFDS is an uncommon genetic disorder, as delineated in this case report, featuring an autosomal dominant mode of inheritance with a favorable prognosis. Case 1 is undergoing management through symptomatic treatment and surgical interventions targeting the presented complaints, and Case 2 and Case 3 are planned for surgery subsequently. Although such genetic disorders are exceptionally rare, their prevalence is notably higher in populations characterized by low socioeconomic status and where consanguineous marriages are customary.
